# Plant-mediated Z-scheme ZnO/TiO_2_-NCs for antibacterial potential and dye degradation: experimental and DFT study

**DOI:** 10.1038/s41598-024-57392-5

**Published:** 2024-04-04

**Authors:** Aayasha Negi, Sumit Ringwal, Minakshi Pandey, Mohamed Taha Yassin

**Affiliations:** 1 Department of Chemistry, IFTM University Moradabad, Lodhipur Rajput, Uttar Pradesh 244102 India; 2 Department of Chemistry, Army Cadet College, Indian Millitary Academy, Dehra Dun, 248007 India; 3 Department of Chemistry, Govind Ballabh Pant University of Agriculture and Technology, Pantnagar, 263145 India; 4 Botany and Microbiology Department, College of Science, King Saud University, 11451 Riyadh, Saudi Arabia

**Keywords:** Z-scheme heterojunction, ZnO/TiO_2_, DFT, Dye deprivation, Antibacterial investigation, Environmental sciences, Environmental chemistry, Pollution remediation

## Abstract

Efficient separation of electron–hole pairs remains pivotal in optimizing photogenerated carrier functionality across diverse catalytic and optoelectronic systems. This study presents the fabrication of a novel hollow direct Z-scheme photocatalyst, ZnO/TiO_2_. A thorough analysis encompassing various techniques such as Ultraviolet–Visible Spectroscopy (UV–Vis), X-ray Diffraction (XRD), Transmission electron Microscopy (TEM), Fourier Transform Infrared Spectroscopy (FT-IR), Thermogravimetric Analysis (TGA), and Energy-Dispersive X-ray Spectroscopy (EDX) provided detailed insights into the complex material characteristics of the ZnO/TiO_2_ heterojunction catalyst. The findings revealed coexisting anatase TiO_2_ and wurtzite ZnO phases, each retaining distinct attributes within the nanocomposites (NCs) structure. The study showcased the photocatalytic efficacy of ZnO/TiO_2_-NCs in decomposing Methylene Blue and Acridine Orange under UV irradiation, correlated with their underlying structures. Enhanced degradation of these dyes resulted from the establishment of a direct Z-scheme heterojunction between ZnO and TiO_2_. Employing Density Functional Theory (DFT) using Quantum ESPRESSO, this research analyzed phase diagrams and band structures, elucidating electronic properties and structural correlations. The study characterized a ZnO/TiO_2_ composite, revealing a band gap of 3.1–3.3 eV through UV–Visible spectroscopy and confirming its formation without impurity phases via XRD analysis. TEM and EDX showed uniform element dispersion (Zn: 27%, Ti: 29.62%, C: 5.03%, O: 38.35%). Computational analysis using DFT indicated a reduction in stable phases with increasing temperature. Enhanced dye degradation was observed (MB: 88.9%, AO: 84%), alongside significant antibacterial activity. In the future we predict that research will focus on development of scaled up production and photocatalytic activity through surface modification, while unveiling mechanistic insights and environmental applicability for multifunctional use in water treatment and antibacterial applications, leading to further advancement of the field.

## Introduction

With the ongoing and rapid global demographic and economic growth, ensuring access to clean water is becoming increasingly vital and challenging. Anticipating potential issues that may arise in the future, the provision of clean water has become a fundamental imperative. In light of this, the removal of contaminants from freshwater sources and the treatment of residual water have become crucial strategies to preserve the natural balance of the biosphere^1,2^. Indeed, traditional water treatment methods like coagulation, flocculation, sedimentation, filtration, and disinfection have been effective in eliminating many common pollutants from water. However, these techniques have their limitations when it comes to dealing with microscopic-scale persistent or emerging micro pollutants. Substances such as antibiotics, pesticides, and certain persistent dyes fall into this category, and they pose a challenge because they either cannot be effectively treated using traditional methods or exhibit reduced efficiency in their removal. As a result, there is a growing need to explore and implement advanced treatment technologies to address the removal of such persistent and emerging micro pollutants from water sources effectively. These ongoing contaminants are well-known for harming our biosphere^3^. Numerous conventional and cutting-edge treatment methods have been examined for the removal of persistent organic pollutants, but semiconductor heterogeneous photocatalysis has received the most attention due to its outstanding ability to completely degrade or even mineralize the organic pollutants with a significantly high concentration of photocatalysts.

Z-scheme heterojunction photocatalysts have recently gained significant attention from researchers due to their capacity to maintain higher oxidation and reduction rates compared to conventional type-II heterojunction materials^4^. These photocatalysts involve a distinctive "Z" shaped pathway for photogenerated charge carriers. This pathway is established by combining a reduction semiconductor with an elevated conduction band (CB) position and an oxidation semiconductor with a lower valence band (VB) position^5,6^. Under light exposure, photoexcited electrons from the higher CB of the reduction semiconductor interact with photoinduced holes from the lower VB of the oxidation semiconductor. This results in the accumulation of excited electrons in the positive VB and photoinduced holes in the negative CB, thus enhancing reduction and oxidation capabilities. Notably, TiO_2_ is recognized for its effectiveness in decomposing organic pollutants, attributed to its non-toxic nature, substantial activity, stability, and cost-efficiency. However, the rapid recombination of photo-excited electron–hole pairs limit its photocatalytic efficiency. Incorporating TiO_2_ into Z-scheme heterojunction structures offers a promising strategy to address this limitation. By pairing TiO_2_ with another semiconductor of complementary energy band positions, charge carrier recombination can be minimized. This integration facilitates efficient charge transfer between the semiconductors, enhancing oxidation and reduction reactions. Z-scheme heterojunction photocatalysts thus provide an innovative route to enhance TiO_2_’s photocatalytic capabilities by optimizing charge carrier separation and utilization^7,8^.

ZnO, an attractive n-type semiconductor with photocatalytic performance comparable to TiO_2_, presents added benefits including non-toxicity and strong physical and chemical stability. In our investigation, a noteworthy revelation emerged: the direct formation of a Z-scheme photocatalytic arrangement between TiO_2_ and ZnO. This innovative photocatalyst system capitalizes on the ZnO conduction band (CB) to enhance reduction potential in photo-induced electrons and leverages the TiO_2_ valence band (VB) to heighten oxidation potential in photo-induced holes. Consequently, this Z-scheme configuration outperforms conventional type-II heterojunction photocatalysts, marking a significant advancement in photocatalytic properties^9^.

In this study, we successfully synthesized ZnO/TiO_2_ direct Z-scheme heterojunction photocatalysts using environmentally friendly methods, ensuring the eco-friendliness of the resulting photocatalysts. However, a new challenge has emerged alongside the effluent recycling process: the need to address the harmful effects of microorganism infection.The "Green Method," which creates NCs using plant resources, has found a thorough answer to this unsolved issue. Since green methods are economical, chemical-free, and environmentally benign, they concentrate on generating the required output without the use of dangerous intermediate^10,11^. The *Taraxacum officinale radix*, commonly known as dandelion belongs to the Asteraceae, Cichorioideae family. The dried roots of dandelion are used in various forms such as medicinal teas, coffee substitutes, and capsules. Among its constituents, taraxacoside, a sesquiterpene lactone glucoside, and p-hydroxyphenylacetic acid were initially identified together as an acylating acid within a sugar ester^12^. Dandelion is believed to possess a choleretic effect, attributed to the presence of compounds like taraxinic acid 1′-glucosyl ester, 11,13-dihydrotaraxinic acid l′-glucoside, and two germacranolide acids esterified with glucose in the root composition. Additionally, the roots of dandelion contain notable quantities of the polysaccharide inulin and the sterol taraxasterol derived from two lupeol isomers^13–15^. Presented below is a table highlighting the selection of plants reported in the synthesis of ZnO/TiO_2_ nanocomposites, along with their respective antibacterial efficacy and the percentage of dye degradation achieved.

**Table:** 

Plant used	Antibacterial potential against	% Degradation of methylene blue	% Degradation of acridine orange	Ref
Aloe vera	*E. coli, S. aureus*	85%	80%	^16^
Neem	*S. aureus, P. aeruginosa*	78%	70%	^17^
Green tea	*E. coli, K. pneumoniae*	60%	55%	^18^
Lemon grass	*S. aureus, P. aeruginosa*	90%	85%	^19^
Basil	*E. coli, S. aureus*	75%	70%	^20^

The leaves of *Taraxacum officinale radix* (TOR) exhibit proficient chelating properties, making them effective agents for binding certain ions. This study contributes to the existing body of knowledge on the utilization of plant extracts, specifically TOR, in conjunction with ZnO/TiO_2_. The investigation involves a comprehensive analysis of the physical characteristics of ZnO/TiO_2_ compounds through multiple methodologies. Furthermore, the study delves into the evaluation of their potential for photodegradation and antibacterial activities. This research expands our understanding of the applications and capabilities of ZnO/TiO_2_ photocatalysts when combined with plant-based extracts such as TOR.

This study breaks new ground by exploring the potential of a plant-mediated Z-scheme ZnO/TiO_2_ photocatalyst, a novel approach that harnesses the power of natural compounds for enhanced antibacterial activity and dye degradation. The integration of experimental analyses and DFT insights adds a unique dimension, providing a comprehensive understanding of the catalyst's performance and mechanisms. Unlike previous reports, this approach showcases enhanced photocatalytic efficiency and antibacterial properties through synergistic plant-mediated effects, marking a significant advancement in the field of photocatalysis.

## Experimental section

### Plant collection

**Figure 1:**
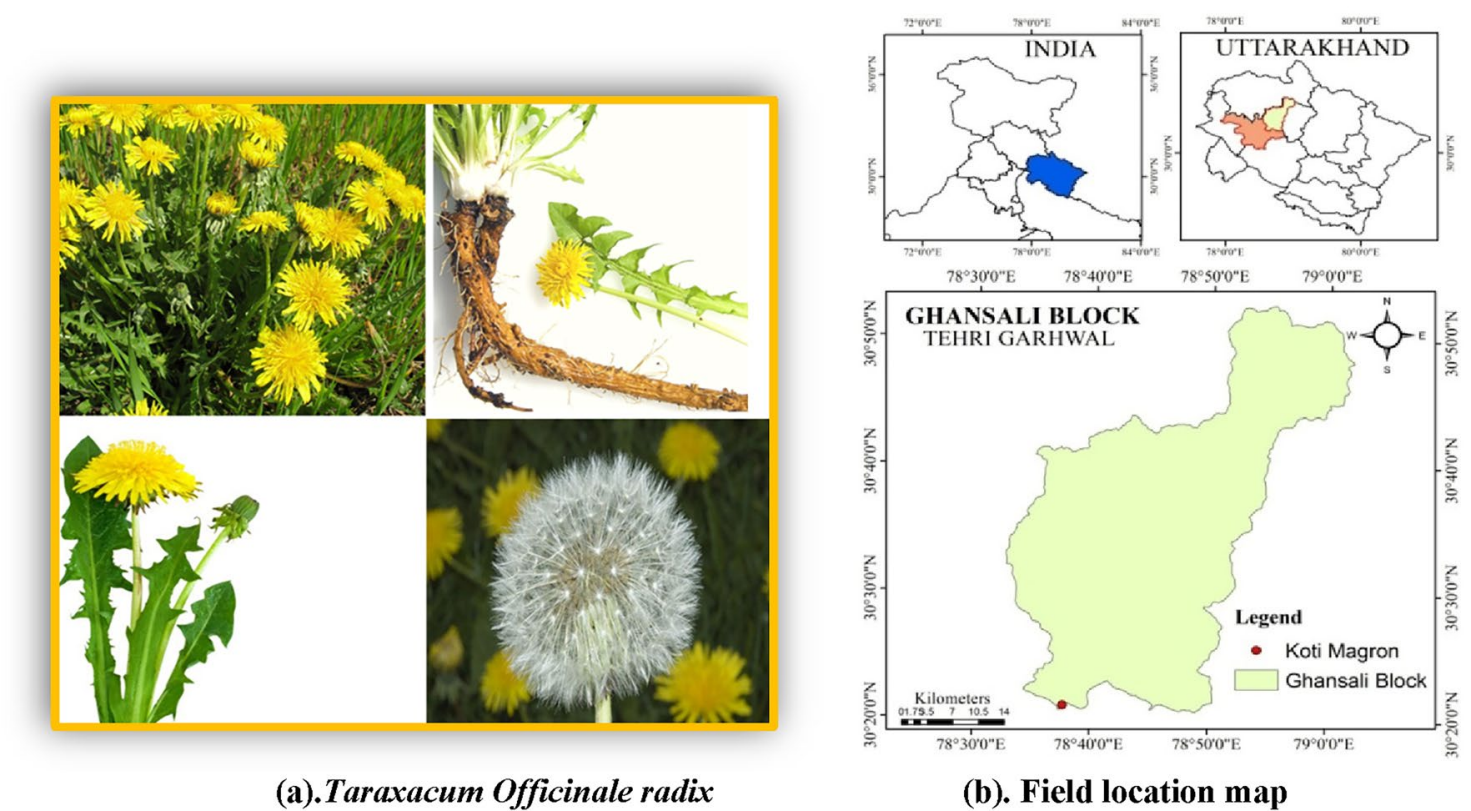
(**a**) *Taraxacum Officinale radix*. (**b**) Field location map.

The TOR Fig. 1a. Foliage was gathered from Tehri Garhwal Uttarakhand, India. As shown in Fig. 1b. During the course of the investigation, double-distilled water was used. "The study conducted on Taraxacum officinale radix adheres to the established guidelines and regulations of H.N.B. Garhwal University, including the rigorous protocols outlined by the esteemed Department of Botany and its herbarium collection."

### Reagents required

Zinc acetate (Zn (CH_3_COO)_2_·6H_2_O) Sigma Aldrich, Titanium dioxide (TiO_2_) (Merck), NaOH, Methylene blue (C_16_H_18_ClN_3_S) Acridine orange (C_17_H_19_N_3_) were acquired from Fischer Scientific. The experiment employed double-distilled water throughout.

### Procured bacterial strains

*Staphylococcus aureus* (MTCC-1144), *Klebsiella pneumoniae* (MTCC-4030), and *Pseudomonas aeruginosa* (MTCC-2474) bacterial strains were sourced from Indian Institute of Microbial Technology (IMTECH) Chandigarh India.

### Statistical analysis

The collected data was presented in the tabulation form. Statistical analysis presents mean values and standard deviations using SPSS 16.0, performing one-way analysis of variance, and comparing means with Duncan tests at a significance level of p ≤ 0.05.

### Methodology followed

#### Plant extract preparation

The procedure commenced with the meticulous cleansing of freshly harvested roots using water, effectively eliminating any adhering impurities. Following this cleaning phase, the roots underwent air-drying to ensure the complete eradication of residual moisture. To facilitate this, the dried roots were subjected to finely grinding using a mechanical grinder, resulting in a finely powdered form that was subsequently employed across all conducted studies.

The preparation of various concentrations of the root extract solution (3%, 5%, 7%, and 10% w/v) followed a systematic approach. Initially, 250 mL of distilled water was meticulously mixed within a 500 mL Erlenmeyer flask. This solution was then meticulously heated to a temperature of 60 °C and meticulously sustained at this specific temperature for a designated period of 30 min. Upon the conclusion of the heating process, the extract was given the necessary time to cool down. Subsequently, the cooled extract was subjected to filtration through Whatman no.1 filter paper, effectively removing any particulate matter^21,22^. The resulting refined concentrate was aptly preserved at a temperature of 4 °C, ensuring its readiness for future investigations and analyses.

#### Synthesis of ZnO/TiO_2_

**Figure 2:**
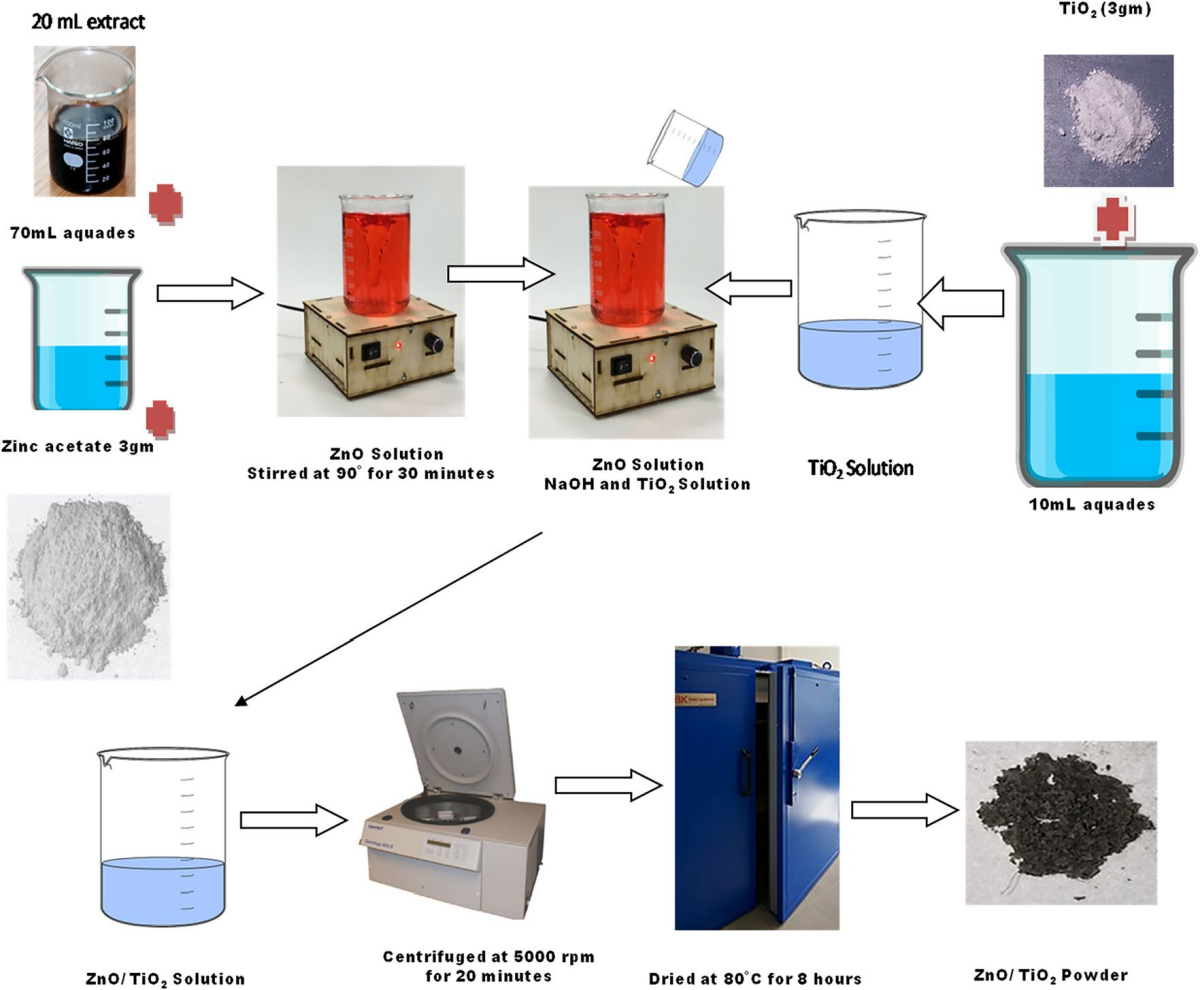
Schematic Presentation of formation of ZnO/TiO_2_.

The obtained filtrate was utilized in the synthesis of ZnO/TiO_2_, and a detailed description of the synthesis processes can be found in Fig. 2. To prepare the solution, 20 mL of TOR leaf extracts, 70 mL of distilled water, and 3 gms of Zn(CH_3_COO)_2_∙2H_2_O were then combined. The solution was stirred at a constant temperature of 90 °C. After 30 min of heating, TiO_2_ was introduced into the zinc oxide solution. For the TiO_2_ solution, 3gms of TiO_2_ powder were mixed with 10 ml of distilled water, and the resulting mixture was homogenized for 5 min using a magnetic stirrer set at a constant speed of 200 rpm. Gradual addition of NaOH followed, with careful drops being introduced until the pH of the solution reached 7, maintaining neutral pH ensures optimal reaction conditions and controlled precipitation during the formation of the composite.The mixture was then subjected to continuous heating and stirring overnight, resulting in the formation of a paste-like consistency. The sample paste was subsequently dried in an oven for 8 h to obtain ZnO/TiO_2_ powder. The powder was then subjected to centrifugation at 5000 rpm for 20 min^22,23^.

### Sample characterization

The biosynthesis process of ZnO–TiO_2_ NCs was meticulously monitored through the utilization of advanced characterization techniques^24^. High-resolution UV–Visible spectrometry employing an Elico SL spectroscope was employed, encompassing the wavelength range of 200–800 nm. Fourier transform infrared spectroscopy, coupled with Perkin Elmer ES Spectrum 2.0 software, was employed to scrutinize the surface adsorption of functional groups on the biosynthesized nanoparticles. This analysis spanned the range of 450–4000 cm^−1^, with a resolution of 4 cm^−1^.

For structural analysis, a PANalytical X*PERT PRO X-ray diffractometer with CuK radiation of 1.54 Å wavelength was employed to examine the purified powders. Additionally, X-ray diffraction data of the NCs were obtained using a Philips PRO expert diffractometer, utilizing nickel-filtered CuK radiation, operated at a voltage of 40 kV and a current of 30 mA. These methodologies facilitated a comprehensive exploration of the structural attributes of the biosynthesized ZnO–TiO_2_ nanoparticles.

SEM-EDAX analyses involved the examination of samples using a SEM Quanta 200 with an EDAX system. The nanoparticles colloid was deposited onto a carbon grid and dried under low vacuum conditions (10–130 pa), maintaining a voltage of 20 keV. The initial sample scanning was conducted at a magnification of 3000x, followed by focused analysis using the EDAX system to affirm the presence of elements. Furthermore, the NPs samples were placed on a carbon-coated copper grid and scanned using the FEI Tecnai TF20 equipped with a 200 kV FEG source, a ± 70 degrees tilted computer-controlled stage, and a 4 K × 4 K Eagle CCD Camera with a 4-port readout and a 15 μm pixel size. EPU software was employed for in-depth data exploration.

To assess thermal stability, Thermogravimetric Analysis (TGA) was performed using the TGA 2950 Thermogravimetric Analyzer. These intricate characterization techniques collectively enabled an exhaustive examination of the nanoparticle’s morphology, composition, and thermal behaviors.

### Computational study

Our investigation commenced with meticulous preparation of accurate crystal structure details for the orthorhombic phase of ZnO/ TiO_2_. In Quantum ESPRESSO software utilizing density functional theory (DFT) calculations, we explored the energy landscape by applying the PBE (Perdew–Burke–Ernzerhof) exchange–correlation functional and incorporating vander Waals interactions^25,26^. Geometry optimization at 300 K & 700 K using the BFGS algorithm revealed stable atomic configurations. Thermodynamic properties like Gibbs free energies were computed, shedding light on essential characteristics. Equilibrium calculations led to the intriguing formation of a triangular phase diagram, illustrating stability regions and coexistence spaces. Simultaneously, the study delved into the combination of ZnO and TiO_2_, analyzing electronic band structures and their alignment. Crystal models for both were constructed using VESTA based on XRD data obtained parameters. The simulation utilized the GGA-PBE exchange–correlation functional, norm-conserving pseudopotentials, and accounted for electron spin polarization. Geometry optimization employed the BFGS method with a plane wave expansion energy cut-off of 750 eV. Monkhorst–Pack (MP) k-point spacings of 0.125/Å and 0.0125/Å were employed for geometry optimization, band structure, and density of states (DOS) calculations. The choice of method was guided by its suitability for semiconductor systems and the balance between accuracy and efficiency. These comprehensive studies contributed to an enhanced understanding of ZnTiO_3_ and the combined ZnO–TiO_2_ system through advanced calculations and insightful analysis.

## Result and discussion

### Characterization details

#### UV–visible spectroscopy and XRD study

**Figure 3:**
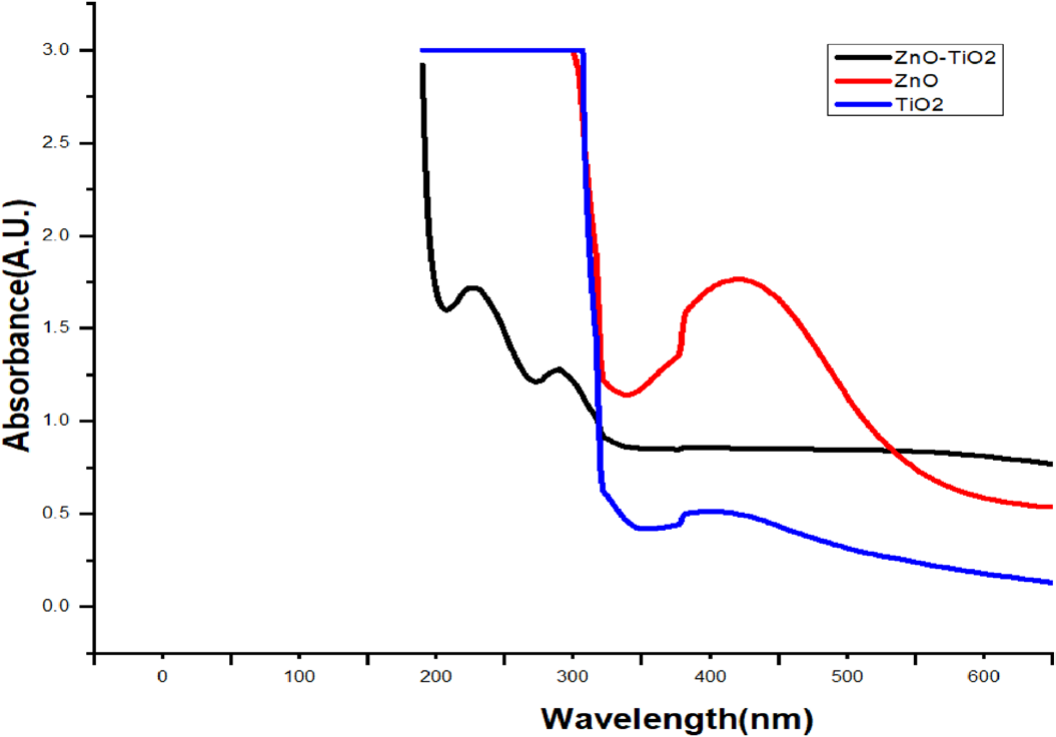
UV–Vis Spectra.

As shown in Fig. 3. Zinc Oxide (ZnO) exhibits a distinctive UV–Vis absorption peak around 368 nm, corresponding to a band gap of approximately 3.37 eV. Titanium Dioxide (TiO_2_), existing in Anatase and phases, showcases absorption peaks at 380 nm, corresponding to band gaps of approximately 3.2 eV and 3.0 eV, respectively. When combined in a ZnO–TiO_2_ composite, the UV–Vis absorption peak appears around 375–390 nm, suggesting an estimated band gap of 3.1–3.3 eV as can be seen. To determine the band gap of the sample directly from the UV–Vis spectrum, the data is converted into Tauc plots using the Kubelka–Munk equation^27^. The equation is expressed as αhv = A(hv–E_g_)^(1/2)^.

In this equation, 'α' represents the absorption, 'A' is the proportional constant, 'h' is the Planck constant, 'v' is the light frequency. This transformation allows the identification of the band gap energy ('E_g_') from the linear portion of the Tauc plot.

**Figure 4:**
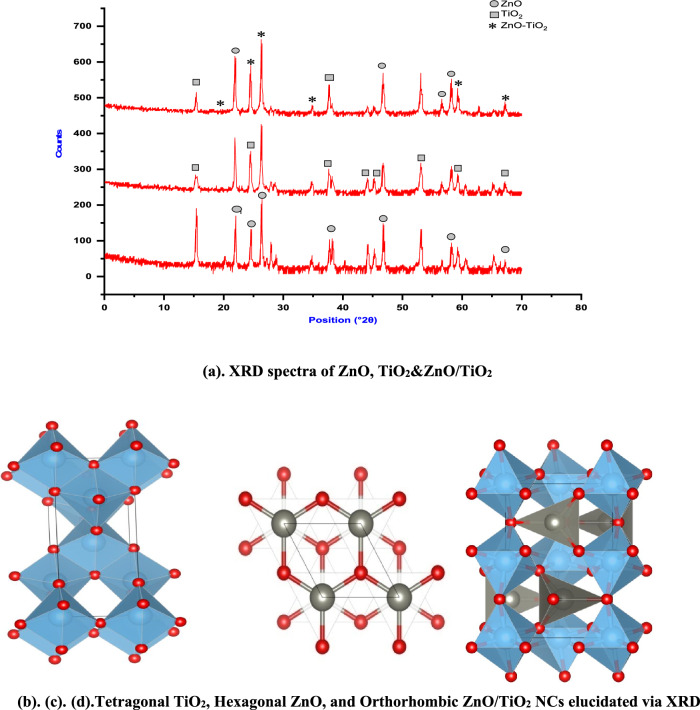
(**a**) XRD spectra of ZnO, TiO_2_&ZnO/TiO_2_. (**b**, **c**, **d**) Tetragonal TiO_2_, Hexagonal ZnO, and Orthorhombic ZnO/TiO_2_ NCs elucidated via XRD.

These values are consistent with previous reports^28–30^. The slight variation in the Eg values further corroborates the successful creation of the TiO_2_/ZnO heterojunction composite^17,18^. The XRD analysis as per Fig. 4a. indicates a significant alignment between the (102) and (103) planes of ZnO and the (200) and (204) planes of TiO_2_. This overlap points to a robust interaction between their lattice structures, conclusively confirming the formation of the ZnO/TiO_2_ composite. Furthermore, minor peaks observed at 2θ angles of 30.14, 42.96, 50.78, and 60.70 correspond to the crystal planes (220), (400), (422), and (440) respectively, and are attributed to the presence of the ZnTiO_3_ phase (JCPDS card No: 39–0190).

The lattice spacing values of 0.35 nm and 0.26 nm, as observed in the XRD patterns of pure TiO2 and ZnO respectively, closely align with those of TiO2 (JCPDS 21–1272) Fig. 4b. and ZnO (JCPDS 89–1397) Fig. 4c. Notably, the absence of any impurity phase affirms the dual-phase composition of the synthesized heterojunction composite. These XRD results provide compelling evidence of the successful creation of the ZnO/TiO_2_ composite, showcasing distinct lattice interactions and the presence of two well-defined phases.

#### FTIR spectroscopy and TGA analysis

**Figure 5:**
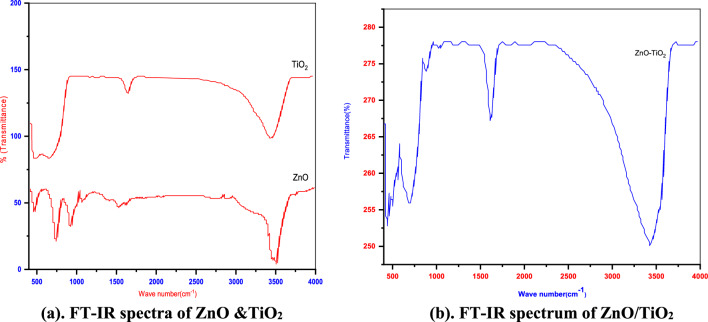
(**a**) FT-IR spectra of ZnO & TiO_2_. (**b**) FT-IR spectrum of ZnO/TiO_2_.

The FT-IR analysis was performed for both the pure TiO_2_ and pure ZnO samples, and the results are shown in Fig. 5a. In the spectrum of the pure TiO_2_ sample, a low-frequency absorption band at 575 cm^−1^ is attributed to the Ti–O–Ti and Ti–O stretching vibrations Turning to the FT-IR spectrum of the ZnO–TiO_2_ composite in Fig. 5b, In the obtained spectra, two broad peaks are distinctly visible at 1630.33 and 3600.5 cm^−1^, corresponding to the O–H stretching vibrations of physically adsorbed water and hydroxyl groups, respectively. Intriguingly, these two peaks exhibit a red shift, with their positions shifting from 1630.33 to 1635.54 cm^−1^ and from 3600.5 to 3475.5 cm^−1^, respectively. Upon closer scrutiny, it becomes evident that the stretching vibration modes of Ti–O–Ti and Ti–O are somewhat attenuated following the incorporation of ZnO nanoparticles into the pore channels of TiO_2_.

This phenomenon finds its root in the displacement of Ti–O bonds of TiO_2_ due to the formation of Ti–O–Zn linkages and Zn–O bonds at the interface between ZnO and TiO_2_.This dynamic results in the creation of a heterojunction structure. Notably, the observed red shift and the observed weakening of specific vibrational modes furnish essential insights into the successful amalgamation and interaction between ZnO and TiO_2_ within the composite material^22,31^.

**Figure 6:**
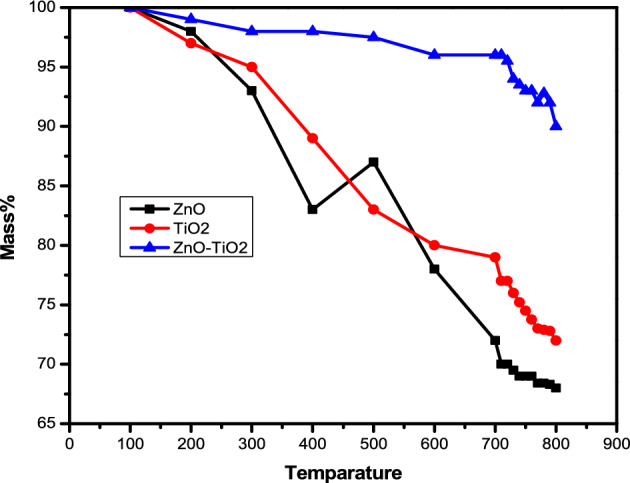
TGA of ZnO, TiO_2_& ZnO/TiO_2_.

The FTIR spectra encompassing the range of 350–4000 cm^−1^ were effectively employed to discern the presence of functional groups and vibrational bonds within the ZnO/TiO_2_ composite. A notable peak at 723 cm^−1^ is attributed to the O–Ti–O bond. Furthermore, various functional groups are discernible in the range of 1000–1100 cm^−1^, notably including the C–H band. At wave numbers of 1447 cm^−1^ and 1665 cm^−1^, distinct vibrational bonds of C=C–C and C=C tensile vibration are apparent, respectively. The O–H bond, pertaining to the vibration of hydroxyl groups present in adsorbed water, is identified at the wavenumber of 3455 cm^−1^^14^. Fig. 6. The provided figure exhibits characteristic TGA (Thermogravimetric Analysis) curves of the biosynthesized ZnO nanoparticles when subjected to a heating rate of 10 °C/min for the sample. The TGA profile reveals a continuous reduction in weight, showcasing three distinct quasi-sharp changes detected at temperatures of 139 °C, 223 °C, and 392 °C, followed by a subsequent nearly constant plateau. This pattern implies that the annealing process above 400 °C ensures the establishment of stable ZnO nanoparticles.

The comprehensive TGA analysis for ZnO/TiO_2_ uncovers three well-defined weight loss regions, contributing to an overall weight reduction of approximately 18%. More specifically, weight losses of roughly 6%, 3%, and 9% are observed in the first, second, and third regions, respectively. The initial weight loss occurring at 700 °C can be attributed to the desorption of adsorbed water from the surface of titania. The weight loss noted at 170 °C is likely linked to the dehydrogenation of –CH_2_–CH_2_–CH_2_–CH_3_ within the as-synthesized ZnO/TiO_2_ coupled with the desorption of crystal water, which signifies the thermal decomposition of any remaining organic groups.

**Figure 7:**
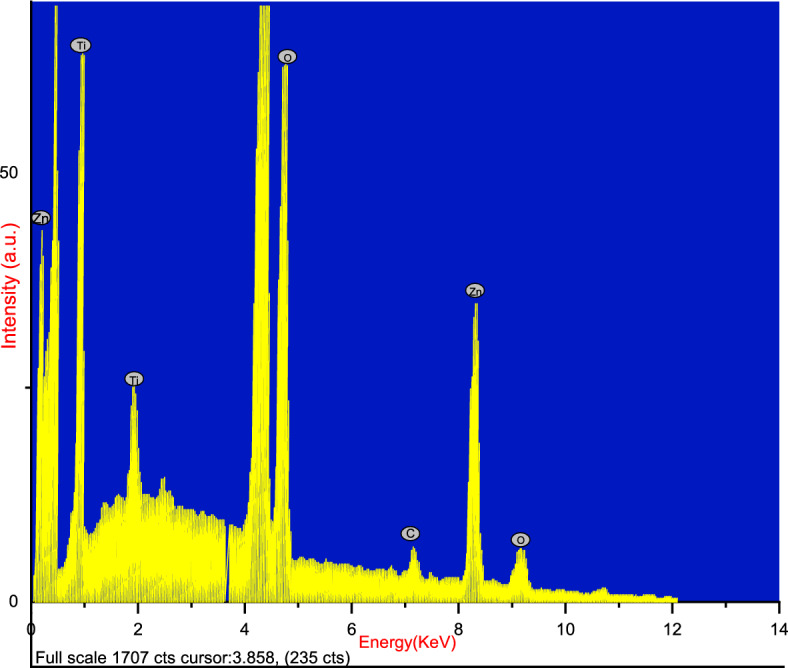
EDX analysis of ZnO/TiO_2_.

#### EDX and TEM studies

**Figure 8:**
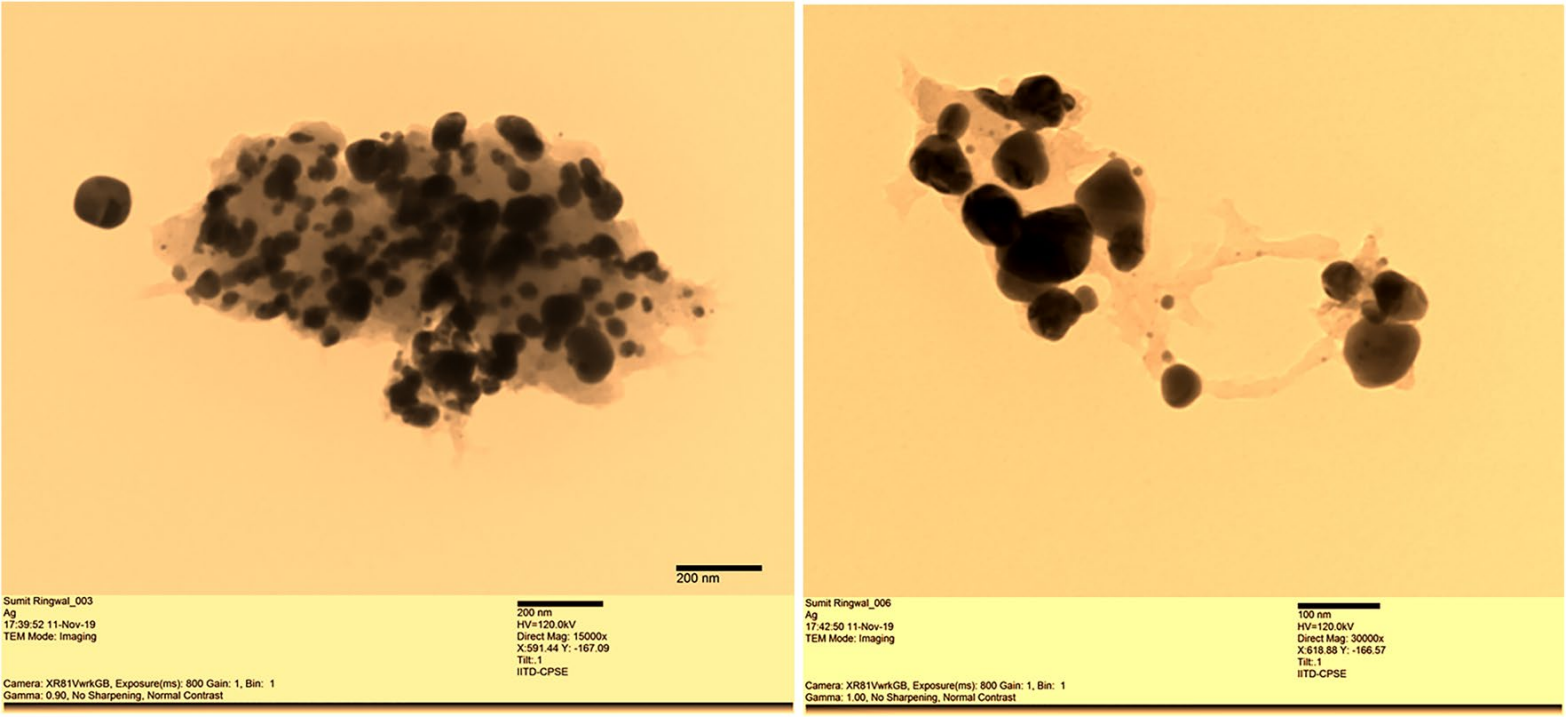
TEM images of ZnO/TiO_2_ at 15,000 × and 30,000 × magnification respectively.

The energy-dispersive X-ray (EDS) analysis, as illustrated in Fig. 7. The TEM images in Fig. 8. unveil the existence of a well-defined macroporous structure within the TiO_2_ component of the sample. Furthermore, the energy-dispersive X-ray (EDS) analysis, as illustrated in Fig. 7. corroborates the concurrent presence of titanium (29.62%), zinc (27%), Carbon (5.03%) and Oxygen (38.35%) elements within the heterojunction composite. Notably, the spatial distribution of these elements is observed to be uniform, signifying a homogeneous dispersion of the heterojunction structure across the entirety of the ZnO/TiO_2_ composite.

### Computational study

#### Compositional phase diagram

**Figure 9:**
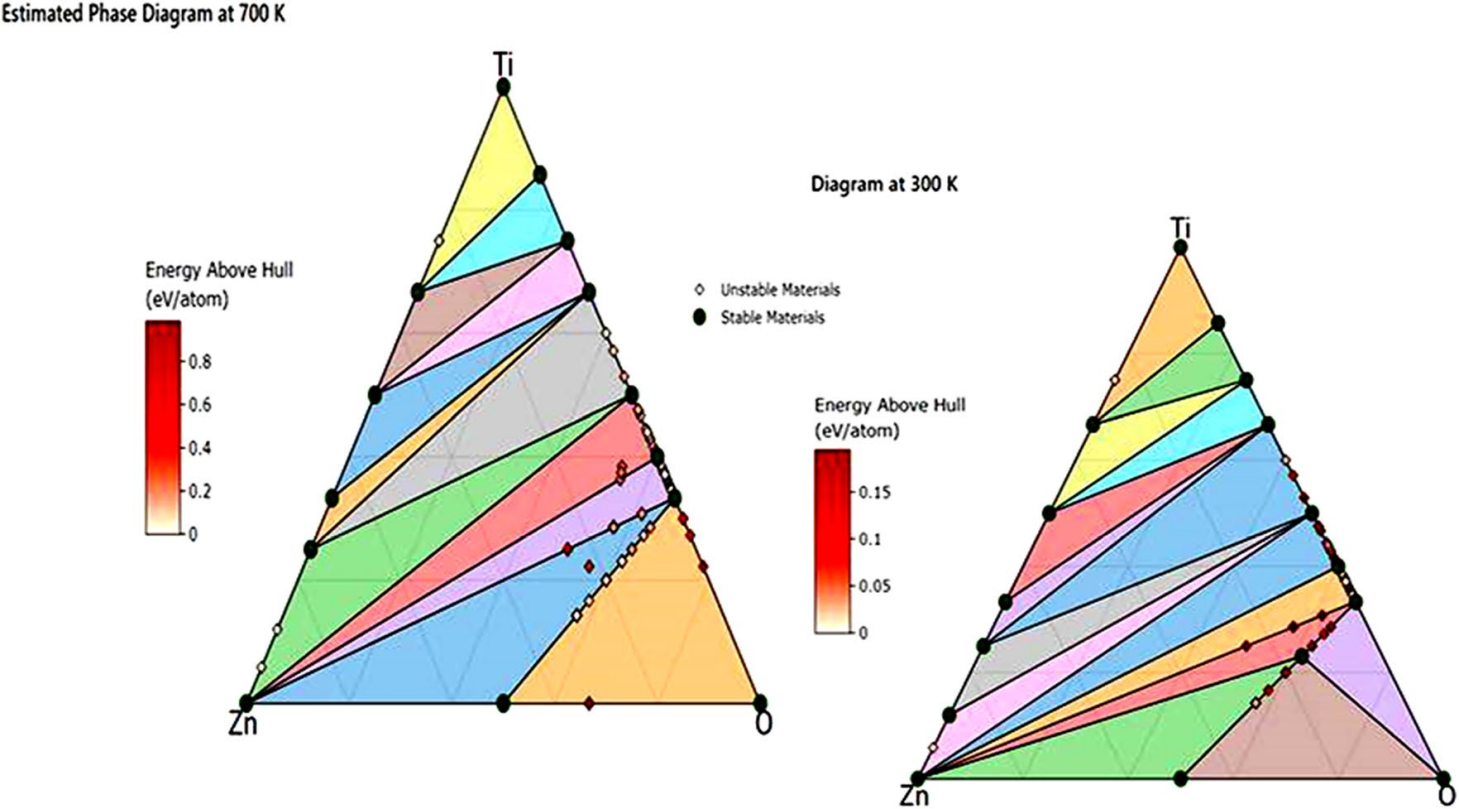
Phase diagram at 700 K and 300 K.

The triangular phase diagram Fig. 9. obtained through Density Functional Theory (DFT) analysis provides valuable insights into the thermodynamic stability and potential phase transitions of a compound at different temperatures. The complexity of the phase diagram at 300 K, with 15 phacets, suggests that the compound demonstrates intricate behavior at this temperature. Each phacet represents a distinct stable or metastable phase that can coexist within the given conditions. Conversely, the phase diagram at 700 K, characterized by 12 phacets, indicates a reduction in the number of stable and metastable phases as temperature increases. This observation implies that certain phases or configurations that were stable at 300 K become less prevalent or unstable at 700 K. This transition could result from changes in molecular interactions, crystal structures, or chemical bonding due to the heightened thermal energy. This phenomenon indicates that certain compounds or phases may become thermodynamically unstable under the conditions of 700 K, leading to their decomposition into other forms or their transformation into more stable phases. The decrease in the number of stable phases highlights the influence of temperature on the equilibrium between different chemical species and their respective phases.

In summary, this triangular phase diagram offers a comprehensive view of the compound's response to temperature variations. The diminishing number of phacets as temperature rises underscores the evolving nature of its stability landscape^32,33^.

#### Band gap and density of state

**Figure 10:**
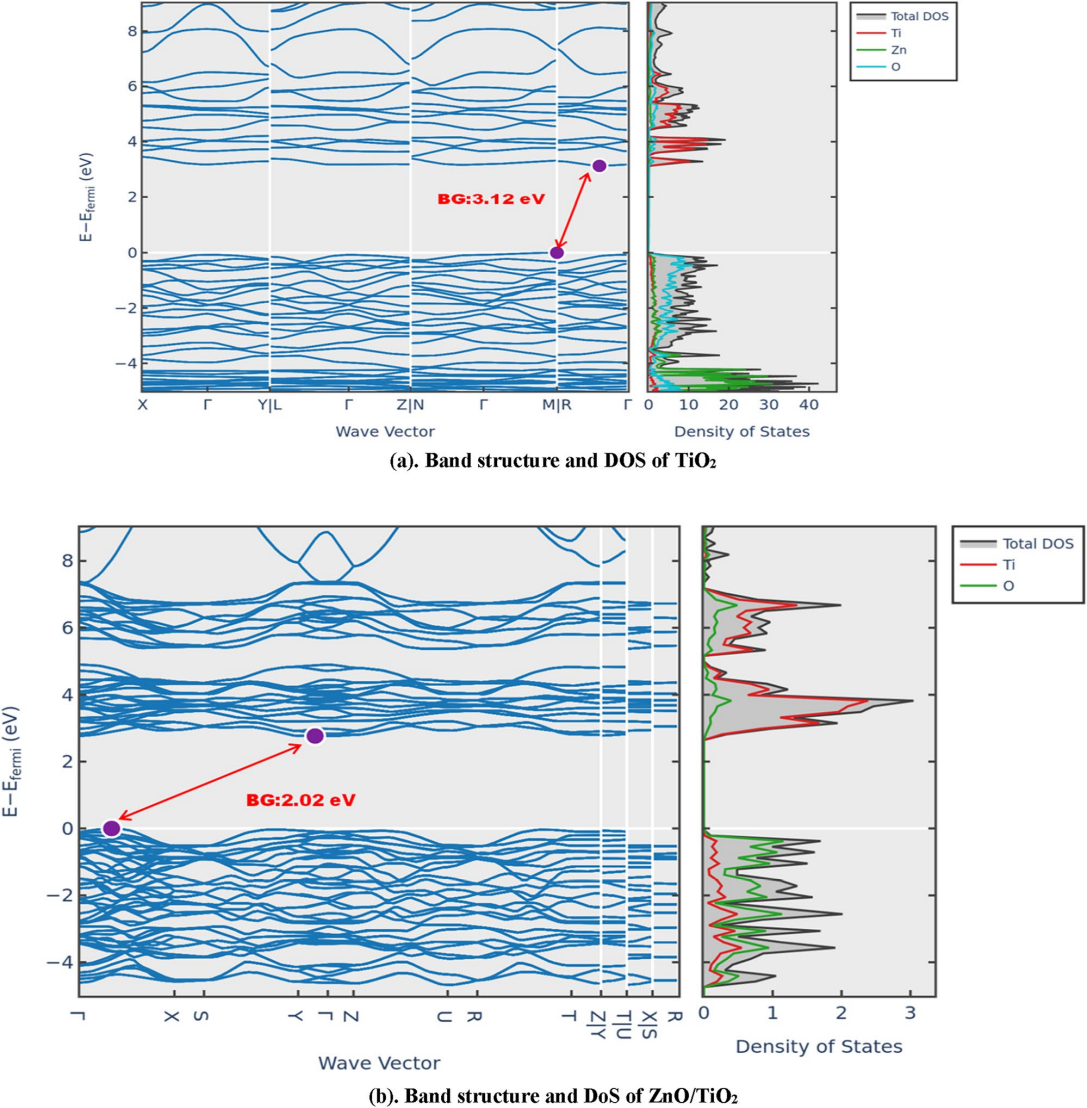
(**a**) Band structure and DOS of TiO_2_. (**b**) Band structure and DoS of ZnO/TiO_2_.

Figure 10a and b. illustrate the band structures of TiO_2_ and ZnO–TiO_2_, respectively. In TiO_2_, the Fermi level resides at the valence band maximum (VBM), whereas in ZnO–TiO_2_, electronic states are denser in both the conduction band minimum (CBM) and VBM, leading to new states near the VBM. These states emerge from impurity levels. Notably, the CBM edge of ZnO–TiO_2_ has shifted compared to the Fermi level, indicating the activity of electrons (e^−^) and holes (h^+^) near the CBM and VBM. The calculated energy gap of TiO_2_ was 2.02 eV while experimental UV–Vis results for TiO_2_ showed 3.3 eV, with theoretical values often lower than experimental ones. In line with the ZnO–TiO_2_ structural model in Fig. 10b, the calculated band gap for ZnO–TiO_2_ was 3.12 eV. These calculated results align well with established experimental data. In TiO_2_, a wide band gap of 2.02 eV makes it useful in applications like photocatalysis. The introduction of ZnO in ZnO–TiO_2_ composite shifts the band gap to 3.12 eV, altering its electronic structure and potentially enhancing its conductivity and optical properties.

#### Dye degradation

The photocatalytic performance of ZnO/TiO_2_ materials in degrading dyes was assessed using a UV–Vis spectrophotometer. During irradiation, the solution exhibited an absorbance spectrum for ZnO/TiO_2_, visible visually or through the absorbance spectra at 400–600 nm. The degradation time for each sample varied due to the charge particles ability to produce hydroxyl and superoxide radicals, crucial in the photodegradation process. The relationship between the percentage of degradation and irradiation times was established, along with corresponding results for samples prepared with different calcination temperatures and ZnO/TiO_2_ concentrations. The absorbance intensity consistently decreased as the time intervals increased, resulting in significant degradation percentages of MB (88.9%) and AO (84%) using ZnO/TiO_2_. These findings demonstrate the excellent potential of ZnO/TiO_2_ composites as photocatalytic materials. The photocatalytic activity was influenced by several factors, including the type of photocatalyst material, crystallite size, and agglomeration level. The photodegradation process is likely influenced by various competing factors^14,34^. Previous reports have shown that reducing the crystallite size leads to an increase in the specific surface area, which enhances the active reaction of the photocatalyst materials.

Upon exposure to visible light, the material undergoes transitions that result in the generation of positively charged holes (h^+^) in the valence band (VB) and highly reactive negatively charged electrons (e^−^) in the conduction band (CB). With the incorporation of ZnO into TiO_2_, the material's absorption edge shifts towards the blue end of the spectrum. This shift indicates the formation of energy levels below the VB, leading to an enlargement of the band gap. This expanded band gap effectively impedes the recombination of electron–hole pairs (e^−^/h^+^), thereby augmenting the material's photocatalytic efficacy.

**Figure 11:**
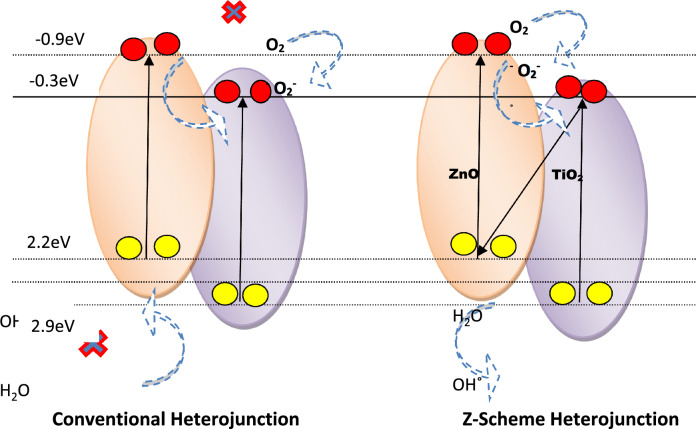
ZnO/TiO_2_ photocatalytic degradation mechanism.

The interface between ZnO and TiO_2_ forms a conventional type-II heterojunction, facilitating the migration of photo-generated electrons from the CB of ZnO to the CB of TiO_2_ under UV irradiation. In parallel, holes migrate from the VB of TiO_2_ to the VB of ZnO, inducing efficient spatial separation of the photoinduced electron–hole pairs. While the accumulated photo-generated holes in the VB of ZnO are unable to oxidize H_2_O to ^•^OH radicals due to the more negative VB edge potential of ZnO compared to the standard redox potential of the ^•^OH/H_2_O pair, and the accumulated electrons in the CB of TiO_2_cannot reduce O_2_ to produce ^•^O_2_^−^ due to the more positive CB of TiO_2_ compared to the standard redox potential of the ^•^O_2_^−^/O_2_ pair, trapping experiments distinctly reveal that O_2_^−^and ^•^OH radicals play a crucial role as the primary active species in the process of photocatalytic degradation, as illustrated in Fig. 11.

**Figure 12:**
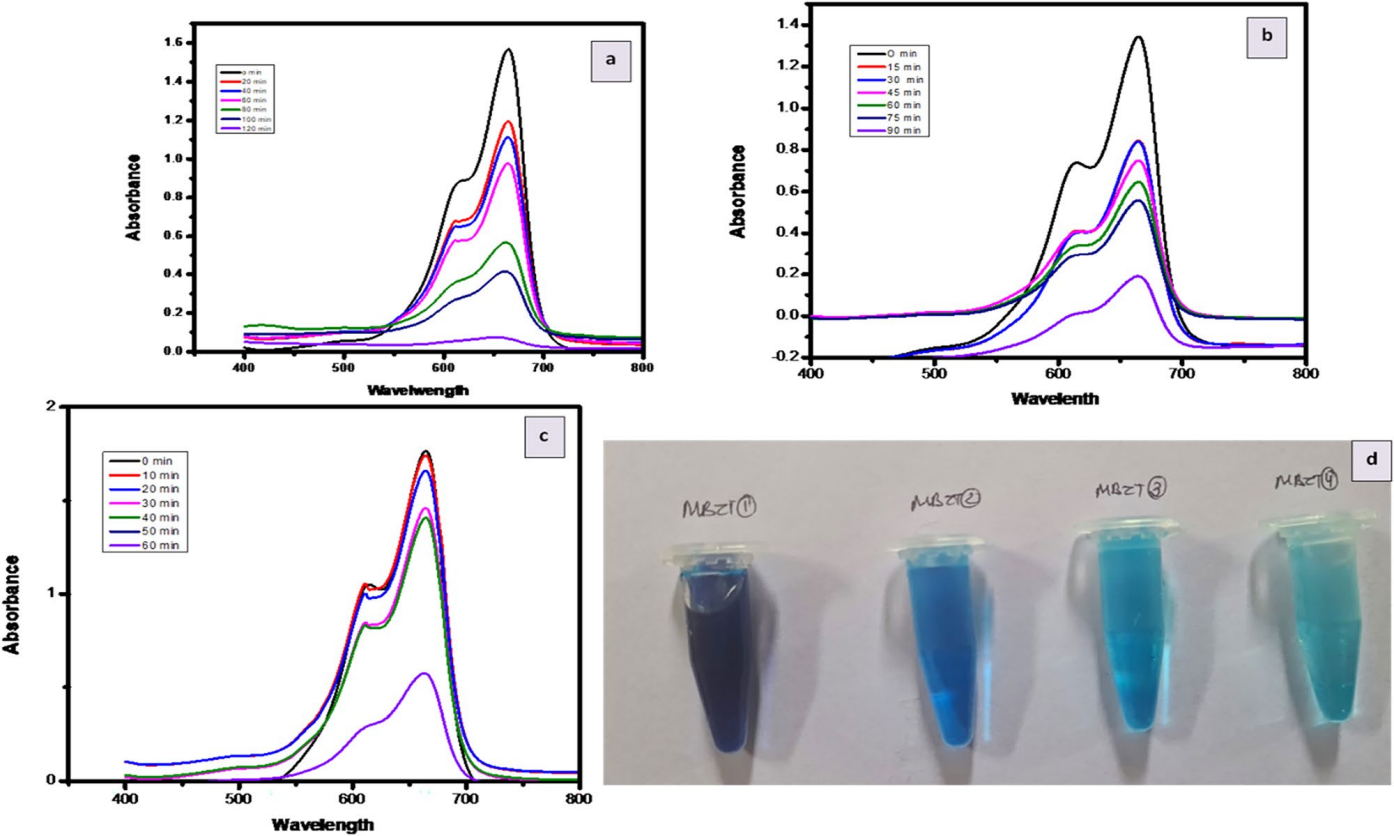
MB degradation by (**a**). ZnO (**b**). TiO_2_ (**c**). ZnO–TiO_2_ (**d**). MB at different interval.

**Figure 13:**
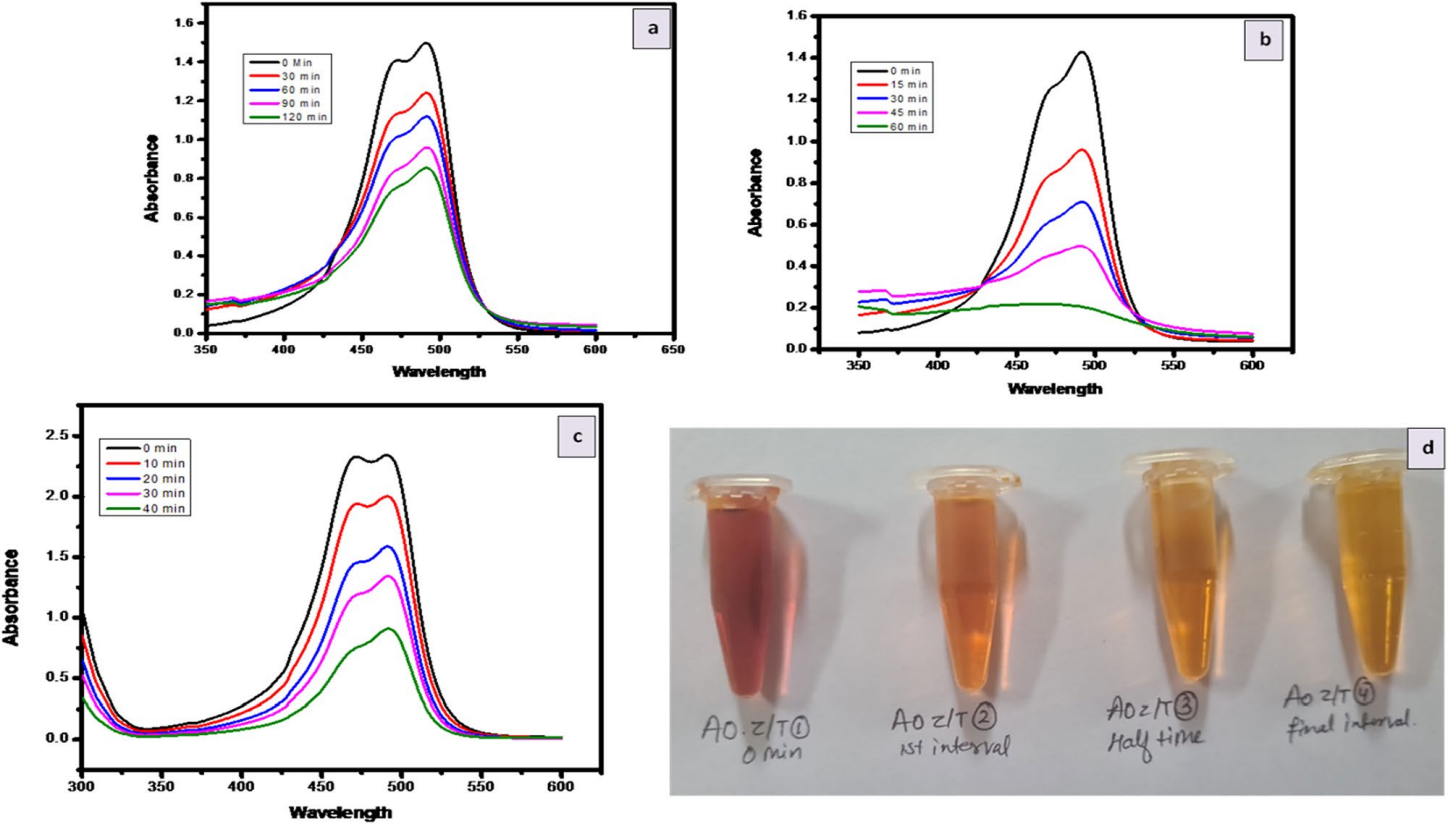
AO degradation by (**a**). ZnO (**b**). TiO_2_(**c**). ZnO–TiO_2_ (**d**). AO at different interval.

The concept of Z-scheme heterojunction provides a comprehensive framework for explaining the experimental findings. When exposed to UV light, photogenerated holes are retained in the valence band (VB) of TiO_2_, while electrons migrate from the conduction band (CB) of TiO_2_ to the VB of ZnO. This arrangement sustains the separation of charges across the junction. Electrons within ZnO, characterized by a potential of − 0.90 eV versus NHE, effectively reduce O_2_ to superoxide ions (^•^O_2_^−^) owing to their negative nature compared to the ^•^O_2_^−^/O_2_ potential of − 0.33 eV versus NHE. Concurrently, the VB of TiO_2_, with a potential of 2.97 eV versus NHE, efficiently oxidizes H_2_O into •OH radicals. This mechanism introduces novel pathways for the generation of radicals. The validation from experiments involving active species trapping and hydroxyl radicals strongly supports the credibility of this ZnO/TiO_2_ Z-scheme photocatalytic mechanism. Photodegradation of dyes Methylene Blue (MB) Fig. 12 and Acridine Orange (AO) Fig. 13. showcased substantial enhancement when utilizing ZnO/TiO_2_ compared to using ZnO or TiO_2_ in isolation. After a 90-min period, the degradation efficiency of MB using ZnO, TiO_2_, and ZnO/TiO_2_ was measured at 43.6%, 52.6%, and 88.9% respectively. Similarly, the degradation of pure AO using ZnO, TiO2, and ZnO/TiO2 reached values of 54.5%, 56.25%, and 84% respectively. The observed photodegradation was influenced by the concentration of the dye.

Furthermore, the degradation rate constants (k) for MB and AO were determined across the various photocatalysts. The k value associated with ZnO was higher than that of pure TiO_2_ and pure ZnO nanoparticles. The optimized ZnO/TiO_2_ hybrid photocatalyst displayed the highest k value in comparison to the pure TiO_2_ and pure ZnO nanoparticles.

#### Antibacterial activity

The mechanism underlying the antibacterial activity of ZnO/TiO_2_ involves the interaction of the particles with bacterial cell membranes. The smaller particle size of ZnO/TiO_2_ results in a larger contact surface area, facilitating enhanced interaction with the bacterial cell membranes compared to pure ZnO or TiO_2_ nanoparticles. This increased interaction leads to the generation of a significant amount of reactive oxygen species (ROS) at the interface between the nanoparticles and the bacterial cells. These ROS, such as hydroxyl radicals and superoxide ions, are known for their potent oxidative abilities, causing damage to the bacterial cell membranes and DNA. Consequently, the bacterial growth is inhibited, ultimately leading to bacterial cell death. The observed higher zone of inhibition for ZnO/TiO_2_ compared to pure ZnO or TiO_2_ against tested pathogens can is due to enhanced generation of ROS, highlighting the synergistic antibacterial effect resulting from the combination of ZnO and TiO_2_^35–37^.

**Figure 14:**
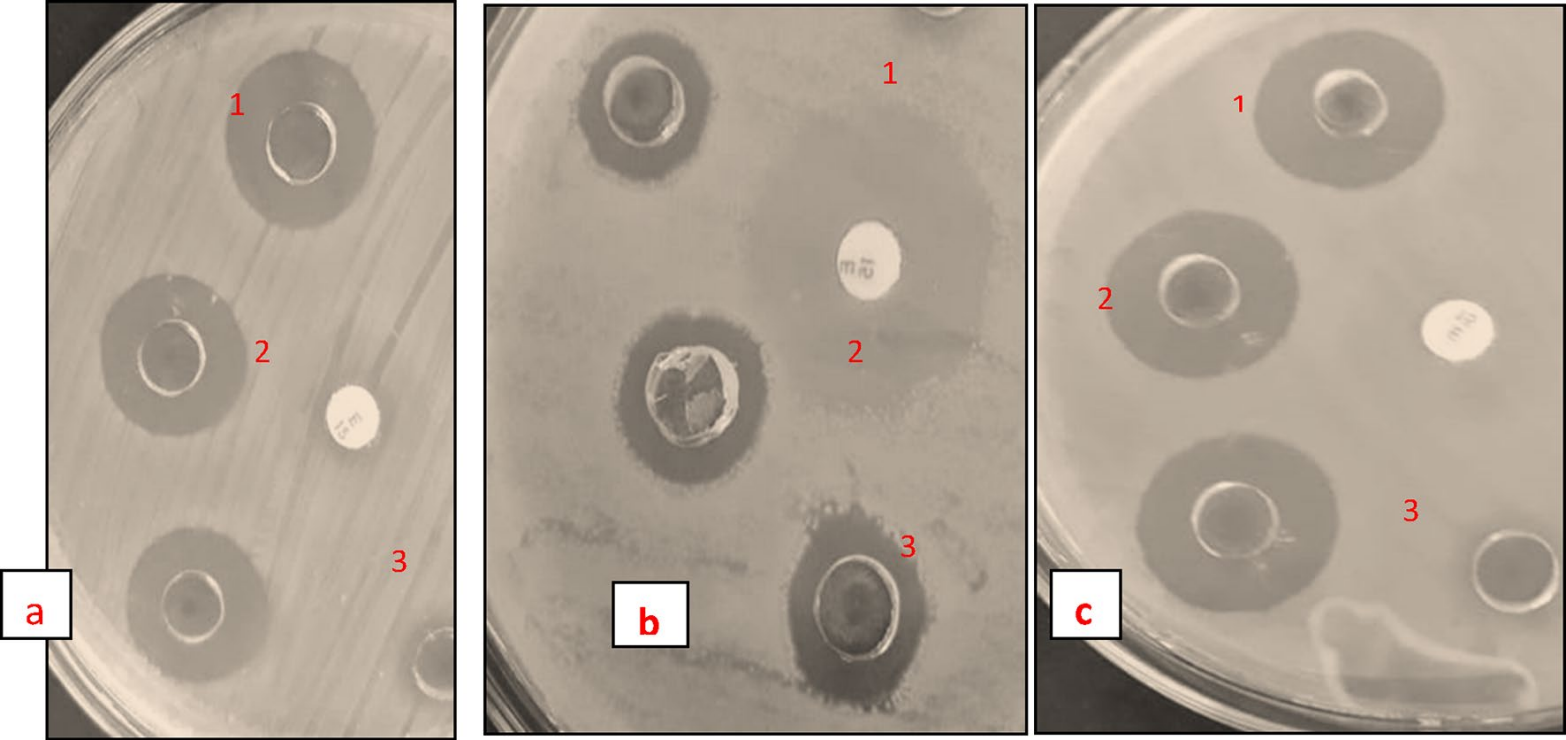
Inhibition zone against various pathogens (**a**) ZnO (**b**) TiO_2_ (**c**) ZnO/TiO_2_.

The agar well diffusion method, previously utilized in other studies, serves as a rapid and effective test to assess the antibacterial activity^38,39^. In this study, ZnO, TiO_2_, and ZnO/TiO_2_ were evaluated against gram-negative pathogens such as *K. pneumoniae* and *P. aeruginosa*, as well as the gram-positive bacteria *S. aureus* (Table 1). The nanoparticles were prepared as a suspension of 150 µg/ml in 0.5% DMSO for testing against the target microorganisms as shown in Fig. 14. The test organisms were evenly spread over the surface of agar plates, which were then incubated at 37 °C to observe the development of zones of inhibition around the wells. The presence and size of these zones of inhibition indicate the antibacterial activity of the nanoparticles.

**Table 1:** Inhibition Zone against various pathogens.

S.N	Pathogen	Control	ZnO	TiO_2_	ZnO/TiO_2_	Erythromycin
1	*k. pneumonia*	–	17.8 ± 0.2	18.2 ± 0.11	19.12 ± 0.14	14.6 ± 0.2
2	*S. aureus*	–	15.7 ± 0.1	16.3 ± 0.21	17.8 ± 0.31	21.3 ± 0.12
3	*P. aeruginosa*	–	16.4 ± 0.16	15.6 ± 0.12	16.6 ± 0.13	13.4 ± 0.43

## Conclusion

In conclusion, the study unveils a series of ZnO/TiO_2_ Z-scheme photocatalysts synthesized through a green process, showcasing enhanced photocatalytic performance. The coexistence of anatase TiO_2_ and wurtzite ZnO phases within the nanocomposites structure contributes to their distinct attributes. Demonstrating significant photocatalytic efficacy, the ZnO/TiO_2_-NCs effectively decompose Methylene Blue and Acridine Orange under UV irradiation, correlating with their underlying structures. Employing Density Functional Theory (DFT) using Quantum ESPRESSO, the research elucidates electronic properties and structural correlations, revealing a band gap of 3.1–3.3 eV through UV–Visible spectroscopy. Moreover, XRD analysis confirms the formation of the ZnO/TiO_2_ composite without impurity phases, while TEM and EDX show uniform element dispersion. Computational analysis using DFT indicates a reduction in stable phases with increasing temperature. Enhanced dye degradation, with Methylene Blue degradation reaching 88.9% and Acridine Orange reaching 84%, along with significant antibacterial activity, underscores the multifunctionality of the ZnO/TiO_2_ photocatalysts. The future direction of research is predicted to focus on scaled-up production, surface modification for enhanced photocatalytic activity, and uncovering mechanistic insights for multifunctional applications in water treatment and antibacterial domains, further advancing the field.

## Data Availability

All data relevant to this study are presented in the manuscript. No additional datasets were generated or analyzed during the current research.
